# Genomic profiling of antimicrobial resistance genes in clinical isolates of *Salmonella* Typhi from patients infected with Typhoid fever in India

**DOI:** 10.1038/s41598-020-64934-0

**Published:** 2020-05-19

**Authors:** Amit Katiyar, Priyanka Sharma, Sushila Dahiya, Harpreet Singh, Arti Kapil, Punit Kaur

**Affiliations:** 1 Department of Biophysics, All India Institute of Medical Sciences, New Delhi, 110029 India; 2 ICMR-AIIMS Computational Genomics Center, Division of I.S.R.M., Indian Council of Medical Research, New Delhi, 110029 India; 3 Department of Microbiology, All India Institute of Medical Sciences, New Delhi, 110029 India

**Keywords:** Computational biology and bioinformatics, Microbiology

## Abstract

The development of multidrug resistance in *Salmonella enterica* serovar Typhi currently forms a major roadblock for the treatment of enteric fever. This poses a major health problem in endemic regions and extends to travellers returning from developing countries. The appearance of fluoroquinolone non-susceptible strains has resulted in use of ceftriaxone as drug of choice with azithromycin being recommended for uncomplicated cases of typhoid fever. A recent sporadic instance of decreased susceptibility to the latest drug regime has necessitated a detailed analysis of antimicrobial resistance genes and possible relationships with their phenotypes to facilitate selection of future treatment regimes. Whole genome sequencing (WGS) was conducted for 133 clinical isolates from typhoid patients. Sequence output files were processed for pan-genome analysis and prediction of antimicrobial resistance genes. The WGS analyses disclosed the existence of fluoroquinolone resistance conferring mutations in *gyrA, gyrB, parC* and *parE* genes of all strains. Acquired resistance determining mechanisms observed included *catA1* genes for chloramphenicol resistance, *dfrA7, dfrA15, sul1* and *sul2* for trimethoprim-sulfamethoxazole and *bla*_*TEM-116*_/*bla*_*TEM-1B*_ genes for amoxicillin. No resistance determinants were found for ceftriaxone and cefixime. The genotypes were further correlated with their respective phenotypes for chloramphenicol, ampicillin, co-trimoxazole, ciprofloxacin and ceftriaxone. A high correlation was observed between genotypes and phenotypes in isolates of *S.* Typhi. The pan-genome analysis revealed that core genes were enriched in metabolic functions and accessory genes were majorly implicated in pathogenesis and antimicrobial resistance. The pan-genome of *S.* Typhi appears to be closed (B_pan_  =  0.09) as analysed by Heap’s law. Simpson’s diversity index of 0.51 showed a lower level of genetic diversity among isolates of *S.* Typhi. Overall, this study augments the present knowledge that WGS can help predict resistance genotypes and eventual correlation with phenotypes, enabling the chance to spot AMR determinants for fast diagnosis and prioritize antibiotic use directly from sequence.

## Introduction

Typhoid fever, a multisystemic disease related to *Salmonella enterica* serovar Typhi (*S*. Typhi) infection is a global threat due to increasing antibiotic resistance to antityphoidal agents in practice^1,2^. Antimicrobial-non-susceptible *Salmonella* infections not only increase disease severity, but also enhance cost of antibiotic treatment and need for hospitalization resulting in economic losses^3,4^. Resistance in typhoidal salmonellae surfaced majorly after introduction of therapy with chloramphenicol^5^. The emergence of multiple drug resistant isolates collectively resistant to chloramphenicol, ampicillin and co-trimoxazole made ciprofloxacin the drug of choice to treat enteric fever. Wide overuse of this drug generated non-susceptible strains due to appearance of mutations in the target enzyme, DNA gyrase and topoisomerase IV^6,7^. Discontinuation of older drug regime removed mutational pressure resulting in reappearance of chloramphenicol susceptible strains. However, the possible generation of plasmid mediated antimicrobial resistance determinants by horizontal transfer has prevented its reuse in clinical practice^8^. Sporadic episodes of extended spectrum beta lactamase genes conferring resistance to current third generation cephalosporins have also emerged^9,10^. Recent emergence of *S*. Typhi isolates resistant to azithromycin in Bangladesh indicated an increasing trend in resistance to azithromycin as compared to the rest of the world^11^. Of great concern is a recent study from Pakistan that reports the outbreak of enteric fever caused by extremely drug resistant (XDR) strains of *S*. Typhi^12^. In this scenario of evolving resistance mechanisms, an examination of the basis of resistance to existing drugs coupled with a genotypic-phenotypic analysis can aid in devising alternative therapeutic targets or strategies for the treatment of multidrug resistant bacterial infections.

Advancements in next generation sequencing technologies have aided whole genome sequencing (WGS) and evaluation of the complete DNA sequence of a bacterium, making it an ideal technique for surveillance^13^. WGS provides definitive genotype information and gives best possible resolution for characterization of an individual organism. Furthermore, strains possessing identical resistance phenotypes conferred by different mechanisms can also be differentiated^14^. Antimicrobial resistance determination by WGS can complement traditional laboratory-based surveillance and provide direct insights into their evolution and transmission from one strain to another. Current genome sequencing methods afford improved and exhaustive data related to the pathogen genotypic characteristics together with the identification of virulence determinants, antimicrobial resistance genes and serotypes whereas conventional antimicrobial susceptibility tests yield the phenotypes of strains. WGS data can help in revealing the antibiotic resistance mechanism^15–18^ for drugs not being tested routinely or where the mechanisms of antimicrobial resistance are not yet identified. Several studies have established a strong antimicrobial genotypes-phenotypes correlation in *E. coli*, *Campylobacter* and non-typhoidal *Salmonella* respectively^16,19,20^.

Antimicrobial resistance is either caused by mutations in chromosomal genes (intrinsic resistance) or by acquisition of plasmid mediated resistance determinants (extrinsic resistance). The intrinsic resistance is mainly due to selection pressure whereas extrinsic genes are acquired by horizontal transfer^21^. The recent trends in the development of antimicrobial resistance (AMR) among *S*. Typhi in India proposed that multidrug resistant (MDR) enteric fever was decreasing in India and being replaced by enteric fever with fluoroquinolone resistant strains^22^. Similarly, hospital-based genomic surveillance for enteric fever in Bengaluru, India using WGS method suggested that large number of isolates showed non-susceptibility to fluoroquinolones^23^. Genome characterization assessment for AMR and pan genome of clinical isolates in endemic countries like India will assist recognition of variations in resistance mechanism and epidemiology leading to better selection of antimicrobial therapy and implementation of appropriate preventive measures. Though typhoidal fever is common across India and specifically in the northern sub-continent, the diversity between the strains is yet unexplored.

In the present study, 133 strains of *S*. Typhi isolated from patients presented with enteric fever to AIIMS hospital during past 24 years were subjected to whole genome sequencing. The pan genome was constructed and the occurrence of plasmid mediated antimicrobial resistance conferring genes and gene mutations determining resistance to anti-typhoidal agents analysed. This was further compared to the phenotypes of each respective strain. The pan genome analysis indicated that the genome remains almost closed. The genetic diversity among *S*. Typhi strains were investigated using the available online resources, such as BacWGSTdb^24^, Center for Genomic Pathogen Surveillance, and Centre for Genomic Epidemiology. These tools offer bacterial typing, rapid classification, source tracing, and phylogenetic relatedness linked to antibiotic resistance genes and clinical data important in a globalized community.

## Result and Discussion

### ***Salmonella*draft genome**

A total of 13,645 contigs varying from 48 to 654 with an average of 102.59 contigs per genome were generated. The average genome size of 4.6 Mb with reads per genome of 47,78,163 base pairs corroborates with other *Salmonella* strains^25^. Likewise, observed averaged G + C content of 51.97% corresponded to other isolates^26^. Functional annotation of the genome predicted a total of 4,607 CDSs, 73 tRNAs, and 1 transfer-messenger RNA. Analysis by Kruskal–Wallis statistical test did not yield any significant differences (P ≤ 0.05) amongst the strains related to their CDS, genome size, GC content, and average gene number. The submission detail for isolates, including statistics of genome assembly and transcript annotation is summarized in Table S1 in Supplementary File 1.

### ***Salmonella*pan- and core- genome**

The pan-genome furnishes a comparative analysis across same species but different strains to determine specific features of that species. The constructed pan-genome offers insights into shared and diverse roles of genes amongst studied isolates. BPGA pipeline with similarity threshold of 90% identified a total of 4185 (70.79%) non-redundant core genes present in all genomes (Fig. S1, Supplementary File 2) and 1273 (21.53%) non-redundant accessory genes occurring in at least one but not present in all genomes. Nearly 454 (7.68%) strain specific unique genes (singletons) with no orthologs in corresponding genomic strains were observed. Overall, the pan-genome with 5912 genes, contains 1.28 times more gene content than each individual *Salmonella* strain. The core to pan-genome ratio disclosed a relatively high degree of conservation with the core-genome comprising 70.79% of the pan-genome. The contribution of 1432 new gene families to the pan-genome from 91 genomes implies that it contained on an average 9.22 new genes per genome. The estimated core-genome size (4185 genes) was higher than previously reported core-genomes of *S.* Typhi (3944 genes)^27^ and *S.* Typhimurium (3846, 3910 and 3890 genes)^28–30^. The predicted core genes from this study were found to be ranging from 4016 to 4545 as determined by various pan-genome tools using *S.* Typhi data^31^. Addition of 42 genomes did not supplement the core genome indicating that further augmentation of genomes would not significantly decrease the size of core genome. Genome-wise statistics revealed a wide variation in the shared accessory genes from 129 (PGS-33) to 523 (PGS-26). The singletons were exclusively present in 76 isolates (57.14%) with the highest in PGS-123 (48 genes). Likewise, 63 genes were exclusively absent in 27 isolates (20.30%) with a maximum of 11 in PGS-65. The presence or absence of gene-families might be attributed to specific roles in emergence of virulence and adaptation to their respective habitats. The pan-genome size and distribution is summarized in Table S2 in Supplementary File 1.

**Figure 1:**
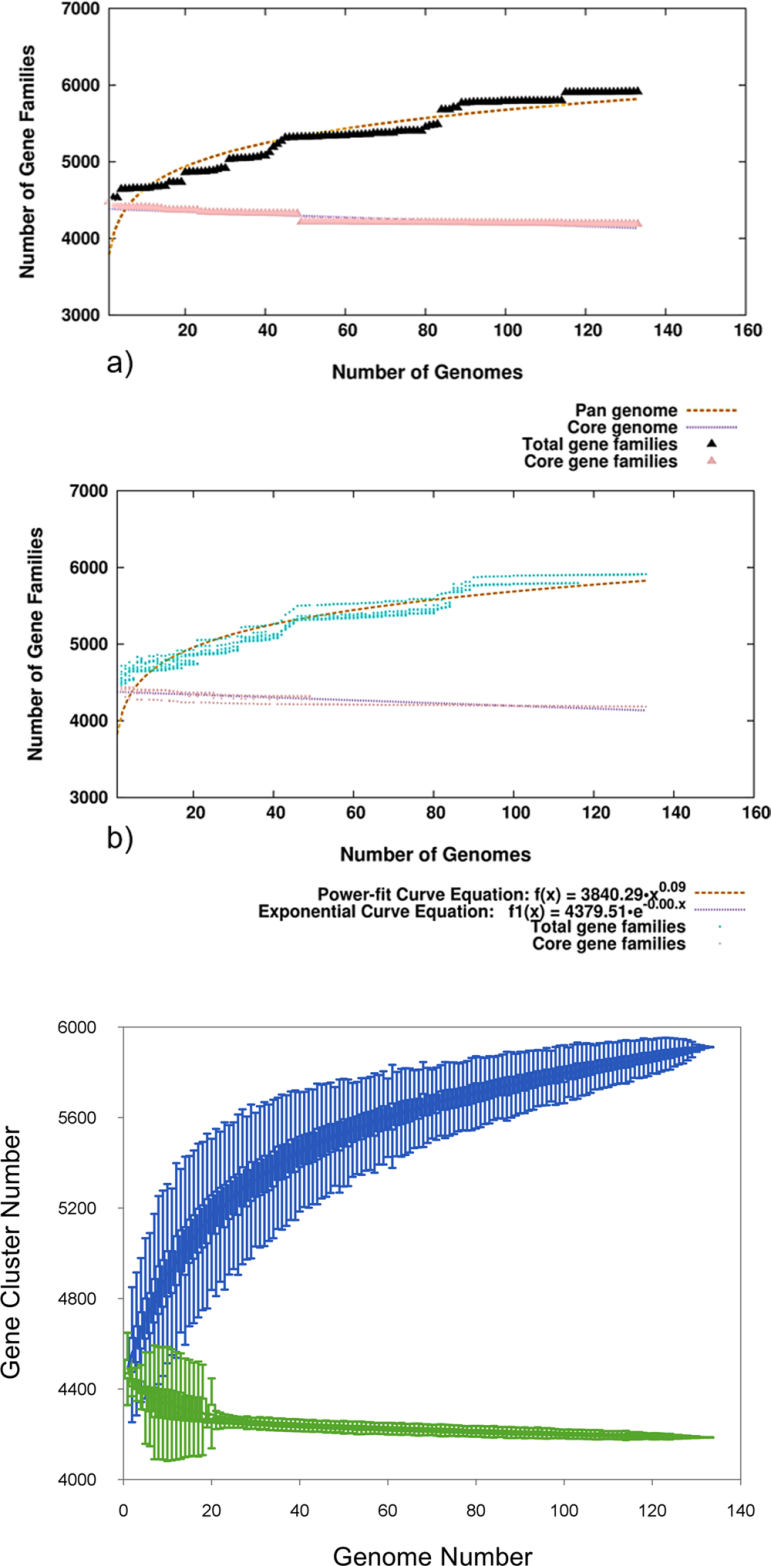
The core-pan genome curve of studied *S.* Typhi genomes. (**a**) Total number of gene families (pan-genome) and shared gene families (core genome) are plotted for a given number of genomes added sequentially. Total gene families are indicated as mustard dashed line while pink dashed line denotes core gene families. (**b**) The mustard dashed line indicates the least-square fit to the power law function f(x) = a.x^b where a = 3840.29, b = 0.0852733. The pink dashed line is the least-squares fit to the exponential decay function f1(x) = c.e^(d.x) where c = 4379.51, d = −0.000429421. (**c**) Pan- and core-genome curve of *S.* Typhi strains disclosed a minor increase in the size of the pan-genome while the core genome appears to almost close.

The closed or open nature of pan-genome evaluates the versatility of the studied strains. The expected gene number in the pan and core-genome was calculated via curve fitting in accordance with Heaps’ law^32^, Y_pan_ = A_pan._ x^B^_pan_ + C_pan_, where y is the size of pan-genome, x is the number of genomes and A_pan_, B_pan_, and C_pan_ the fitting parameters and exponential equations (Y_core_ = A_core. e-_B_core_.x + C_core_), respectively. In this equation, B_pan_ defines whether the pan-genome is closed (B_pan_ < 0 or B_pan_ > 1) or open (0 < B_pan_ < 1)^33^. Total number of gene families (pan-genome) and shared gene families (core genome) are plotted for a given number of genomes added sequentially. The plot indicated a minor expansion of the pan-genome (Bpan = 0.09) while the core genome appeared to extend to almost a closed state as the addition of newer genomes failed to increment any novel gene in the existing genome (Fig. 1). This supports earlier studies demonstrating *S.* Typhi to be a closed pan-genome^34^.

### Functional distribution of genes in pan-genome

**Figure 2:**
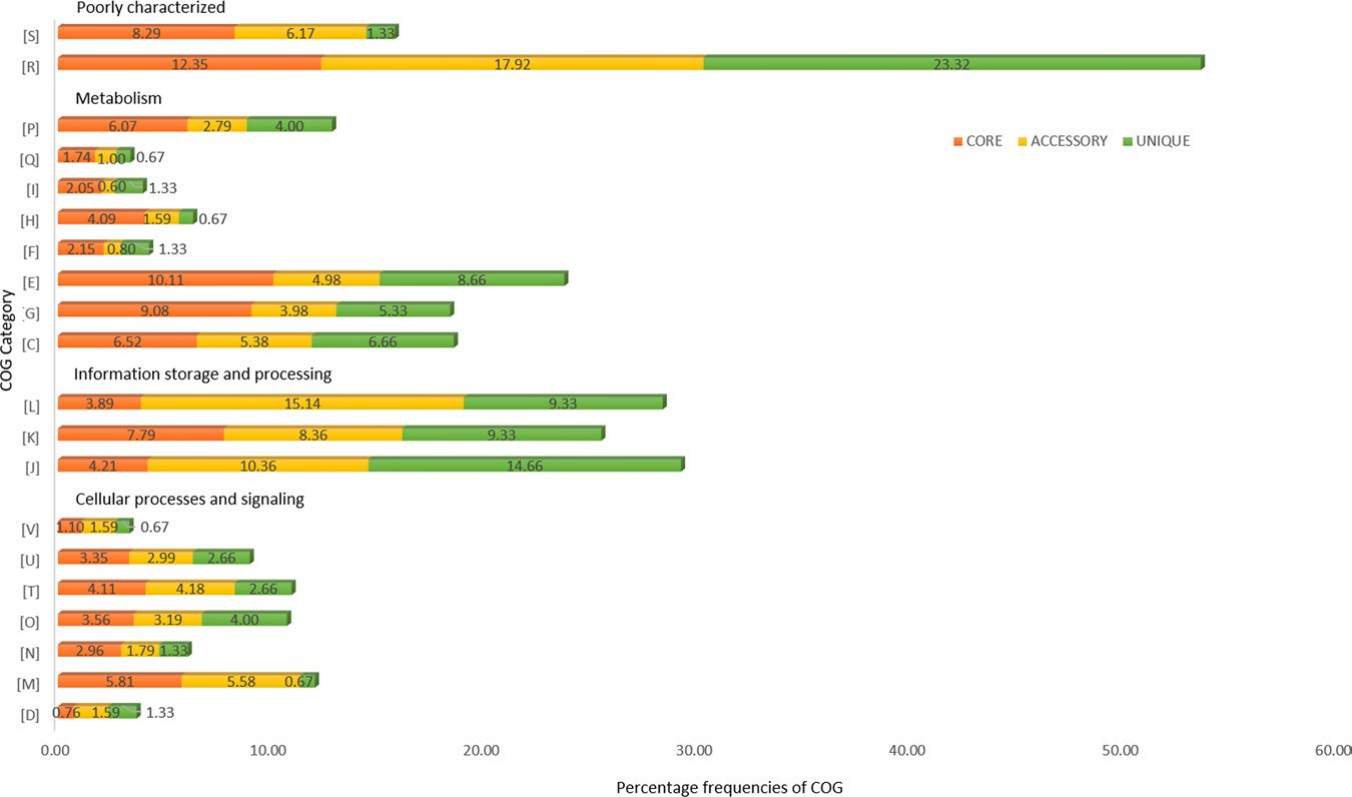
COG functional distribution of pan-genome. The histogram illustrates the predicted functionality of proteins assigned to core (orange), accessory (yellow) and unique (green) genes. The COG families were broadly grouped into four category namely information storage and processing (assigned to the L, K, and J categories), cellular processes and signalling (assigned to the V, U, T, O, N, M and D categories), metabolism (assigned to the P, Q, I, H, F, E, G, and C categories), and poorly characterized (assigned to the S, and R categories). **COG categories:** [**C**] Energy production & conversion; [**D**] Cell cycle control, cell division, chromosome partitioning; [**E**] Amino acid transport & metabolism; [**F**] Nucleotide transport; [**G**] Carbohydrate transport & metabolism; & metabolism; [**H**] Coenzyme transport & metabolism; [**I**] Lipid transport & metabolism; [**J**] Translation, ribosomal structure & biogenesis; [**K**] Transcription; [**L**] Replication, recombination & repair; [**M**] Cell wall/membrane/envelope biogenesis; [**N**] Cell motility; [**O**] Post-translational modification, protein turnover & chaperones; [**P**] Inorganic ion transport & metabolism; [**Q**] Secondary metabolites biosynthesis, transport & catabolism; [**R**] General function prediction only; [**S**] Function unknown; [**T**] Signal transduction mechanisms; [**U**] Intracellular trafficking, secretion & vesicular transport; [**V**] Defense mechanisms.

**Figure 3:**
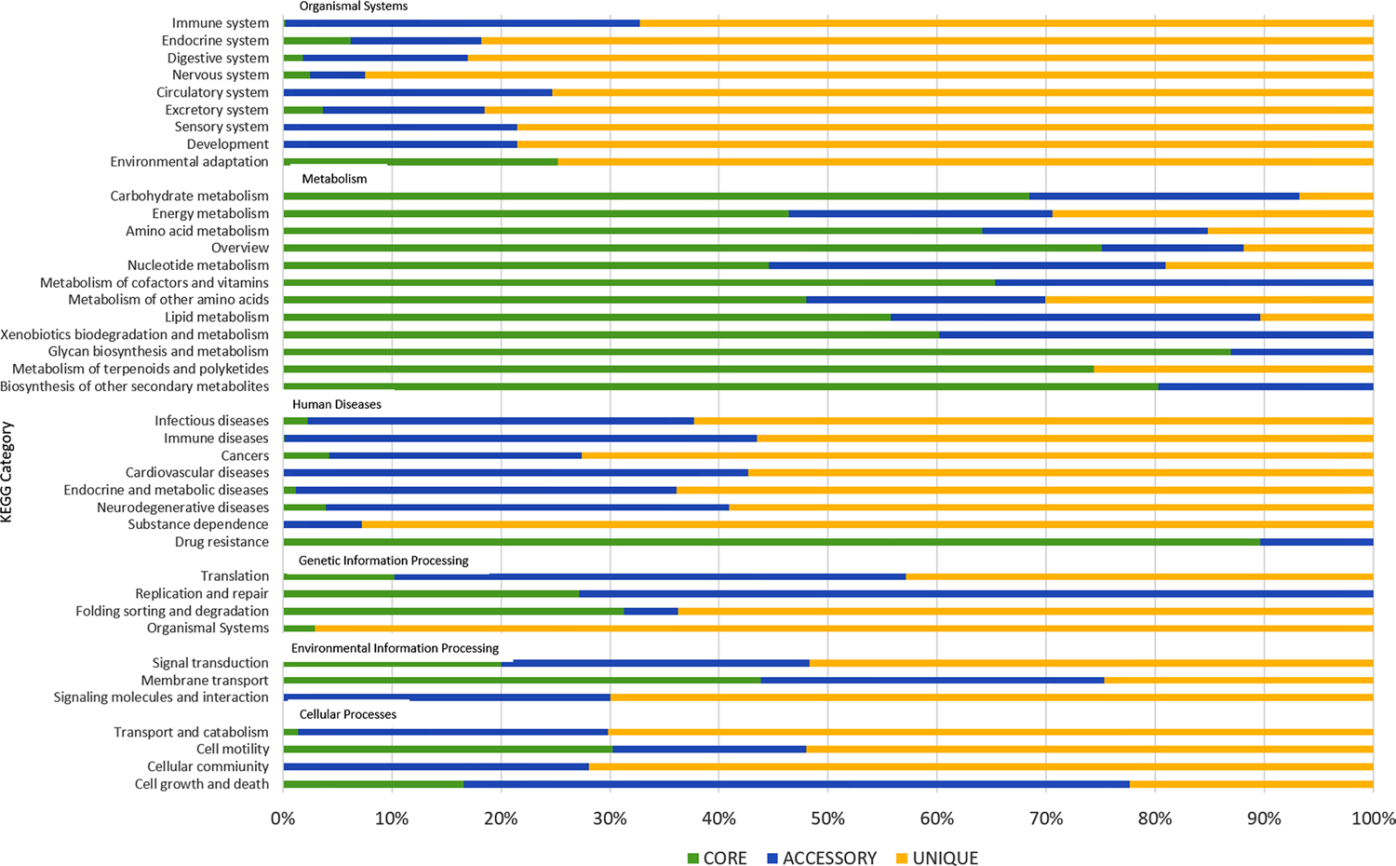
KEGG functional distribution of pan-genome. The graph represent the KEGG functionality of proteins assigned to core (green), accessory (blue) and unique (yellow) genes. The KEGG families were broadly grouped into five category namely cellular processes, human diseases, genetic information processing, environmental information processing, metabolism, and organismal systems.

The pan-genome was further gauged for functional role of the constituent genes. The core, accessory and unique genes were examined for their diverse features by exploring the functional databases COG and KEGG^35–38^. This annotation reflected that the genes are scattered in an assorted range of diverse functional categories throughout the genome. The COG distribution (Fig. 2) predicted the core genome (41.82%) to be predominantly associated with metabolic functions like transport and metabolism of nucleotides, amino acids, coenzyme, carbohydrates, secondary metabolites biosynthesis, lipids and inorganic ions, energy production and conversion and transport and catabolism^39,40^. These categories had some representation in the accessory genome. Some genes were implicated in translation and ribosomal structure mechanism. This analysis suggests that the majority of the core genes are essential for cell survival and necessary for the basic activity of the species. Nearly one-third genes in the accessory (33.86%) and unique (33.31%) genome were primarily involved in basic cellular processes of ‘information storage and processing’, namely translation, ribosomal structure and biogenesis, replication, recombination, repair, and transcription. Though observed in the core genome, these genes were highly represented in the accessory genome signifying inter-species variations. The ‘cellular processes and signalling’ category was enriched among the core genes (21.65%) and depleted in the unique (13.32%) genome. More than one-fifth genes in the core (20.64%), accessory (24.10%) and unique (24.65%) genome were either poorly categorized or uncategorized (Fig. 3). The COG functional distribution of pan-genome is summarized in Table S3 in Supplementary File 1.

The KEGG functional distribution revealed similar overall division of core genes associated with “metabolisms” that accounted for a huge part in core (61.32%) compared to accessory (24.59%), and unique (13.50%) genomes (Fig. 3). Genes were mainly associated with ‘carbohydrate metabolism’, ‘overview’, ‘amino acid metabolism’ and ‘energy metabolism’. A sizeable gene proportion in unique (88%) and accessory (50.55%) genome, compared to the core-genome, participated in ‘human diseases’ related to ‘infectious diseases’, ‘immune diseases’, ‘cancer’ and ‘cardiovascular disease’. Remarkably, 70 genes in the core-genome were associated with pathway ‘drug resistances: antimicrobial’ comprising 38 ko01503: cationic antimicrobial peptide (CAMP) resistance genes, 23 ko01501: beta-lactam resistance genes, and 9 ko01502: vancomycin resistance genes. This suggests that antibiotic resistance genotype plays a vital role in determining *S.* Typhi susceptibility. Overall 2711 (64.78%), 490 (38.49%), and 413 (90.97%) genes in core, accessory and unique-genome, respectively were assigned to a KEGG category. The KEGG functional distribution of pan-genome is summarized in Table S4 in Supplementary File 1.

### Genotypic and Phenotypic resistance to antityphoidal agents

Antimicrobial susceptibility patterns were detected by WGS for resistance determining genes (genotype) and disk diffusion method (phenotype) for first line antibiotics (co-trimoxazole, amoxicillin, and chloramphenicol), fluoroquinolones (ciprofloxacin, and pefloxacin), third generation cephalosporins (cefixime and ceftriaxone) and macrolides (azithromycin) as per CLSI guidelines. All the strains which were collectively resistant to ampicillin, chloramphenicol and co-trimoxazole were considered as MDR (Multiple drug resistant) strains. The relationships between resistance gene content identified from WGS was deciphered with the drug resistance profile for each corresponding clinical isolate (phenotype). None of the 133 strains tested by phenotypic method was found to be susceptible to all antimicrobial agents. Out of the 133 strains, 111 (83.5%; 95% CI, 76.2% to 88.8%) revealed non-susceptibility to one antibiotic and 23 (17.3%; 95% CI, 11.8% to 24.6%) showed non-susceptibility to two or more antibiotics. The antibiotic susceptibility patterns are discussed individually.

### Amoxicillin resistance

**Figure 4:**
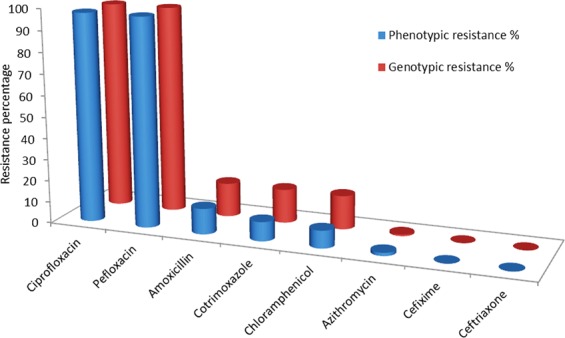
Genotypes-Phenotypes Correlation. Schematic representation of correlation between genotype and phenotype obtained from WGS and disk diffusion method, respectively. Overall correlation of genotypic and phenotypic was 91.83% and 99.06% for sensitivity and specificity, respectively.

The amoxicillin resistance is associated with the presence of beta-lactam genes which were observed in 15.79% (21/133) strains by WGS. The most common beta-lactam resistance gene *bla*_*TEM-1B*_ was observed in 19 strains, *TEM-1* in 16 strains and *bla*_*TEM116*_ in one strain (Table 1; Table S5 in Supplementary File 1). The resistance genes encode for the predominant plasmid-mediated β-lactamases of *Enterobacteriaceae*^41^. Overall, antimicrobial resistance was observed in 12.03% (16/133) isolates by phenotypic method. Earlier reports for amoxicillin resistance in *Salmonella* strains isolated pan-India was 3%^4^. The sensitivity and specificity was 100% and 95.73%, respectively for beta-lactams (Fig. 4; Table 3; Table S7 in Supplementary File 1).

**Table 1:** Antimicrobial resistance profile of 133 *Salmonella* isolates.

Isolate	AMP	AMX	CHL	CIP	PEF	NAL	SMX	TMP	SXT	TCY	AZM	CFM	CTR
PGS-1	0	0	0	0	0	0	0	1	0	0	0	0	0
PGS-2	1	1	1	1	1	1	1	1	1	1	0	0	0
PGS-3	0	0	0	1	1	1	0	0	0	0	0	0	0
PGS-4	1	1	1	1	1	1	1	1	1	1	0	0	0
PGS-5	0	1	1	1	1	1	0	0	0	1	0	0	0
PGS-6	0	1	1	0	0	0	0	0	0	0	0	0	0
PGS-7	0	0	1	1	1	1	0	0	0	0	0	0	0
PGS-8	1	1	1	1	1	1	1	1	1	1	0	0	0
PGS-9	0	0	0	1	1	1	0	0	0	0	0	0	0
PGS-10	0	0	0	1	1	1	0	0	0	0	0	0	0
PGS-11	0	0	0	1	1	1	0	0	0	0	0	0	0
PGS-12	0	0	0	1	1	1	0	0	0	0	0	0	0
PGS-13	0	0	0	0	0	0	0	0	0	0	0	0	0
PGS-14	0	0	0	1	1	1	0	0	0	0	0	0	0
PGS-15	0	0	0	1	1	1	0	0	0	0	0	0	0
PGS-16	0	0	0	1	1	1	0	0	0	0	0	0	0
PGS-17	0	0	0	1	1	1	0	0	0	0	0	0	0
PGS-18	0	0	0	1	1	1	0	0	0	0	0	0	0
PGS-19	0	0	0	1	1	1	0	0	0	0	0	0	0
PGS-20	0	0	0	1	1	1	0	0	0	0	0	0	0
PGS-21	1	1	1	1	1	1	1	1	1	1	0	0	0
PGS-22	0	1	0	1	1	1	1	0	1	0	0	0	0
PGS-23	0	0	0	1	1	1	1	0	1	0	0	0	0
PGS-24	0	0	0	1	1	1	0	0	0	0	0	0	0
PGS-25	1	1	1	1	1	1	1	1	1	1	0	0	0
PGS-26	1	1	1	1	1	1	1	1	1	1	0	0	0
PGS-27	1	1	1	1	1	1	1	1	1	0	0	0	0
PGS-28	0	0	0	1	1	1	0	0	0	0	0	0	0
PGS-29	0	1	0	1	1	1	1	1	1	1	0	0	0
PGS-30	0	0	0	1	1	1	1	1	1	1	0	0	0
PGS-31	0	1	0	1	1	1	0	0	0	0	0	0	0
PGS-32	0	0	0	1	1	1	0	0	0	0	0	0	0
PGS-33	0	0	0	1	1	1	0	0	0	0	0	0	0
PGS-34	0	0	0	1	1	1	0	0	0	0	0	0	0
PGS-35	0	0	0	1	1	1	0	0	0	0	0	0	0
PGS-36	0	0	0	1	1	1	0	0	0	0	0	0	0
PGS-37	0	0	0	1	1	1	0	0	0	0	0	0	0
PGS-38	0	0	0	1	1	1	0	0	0	0	0	0	0
PGS-39	0	0	0	1	1	1	0	0	0	0	0	0	0
PGS-40	0	0	0	1	1	1	0	0	0	0	0	0	0
PGS-41	0	0	0	1	1	1	0	0	0	0	0	0	0
PGS-42	0	0	0	1	1	1	0	0	0	0	0	0	0
PGS-43	0	0	0	1	1	1	0	0	0	0	0	0	0
PGS-44	0	0	0	1	1	1	0	0	0	0	0	0	0
PGS-45	0	0	0	1	1	1	0	0	0	0	0	0	0
PGS-46	0	0	0	1	1	1	0	0	0	0	0	0	0
PGS-47	0	0	0	1	1	1	0	0	0	0	0	0	0
PGS-48	0	0	0	1	1	1	0	0	0	0	0	0	0
PGS-49	0	0	0	1	1	1	0	0	0	0	0	0	0
PGS-50	0	0	0	1	1	1	0	0	0	0	0	0	0
PGS-51	0	0	0	1	1	1	0	0	0	0	0	0	0
PGS-52	0	0	0	1	1	1	0	0	0	0	0	0	0
PGS-53	1	1	1	1	1	1	1	1	1	0	0	0	0
PGS-54	0	0	0	1	1	1	0	0	0	0	0	0	0
PGS-55	0	0	0	1	1	1	0	0	0	0	0	0	0
PGS-56	0	0	0	1	1	1	0	0	0	0	0	0	0
PGS-57	0	0	0	1	1	1	0	0	0	0	0	0	0
PGS-58	0	0	0	1	1	1	0	0	0	0	0	0	0
PGS-59	0	0	0	1	1	1	0	0	0	0	0	0	0
PGS-60	0	0	0	1	1	1	0	0	0	0	0	0	0
PGS-61	0	0	0	1	1	1	0	0	0	0	0	0	0
PGS-62	0	0	0	1	1	1	0	0	0	0	0	0	0
PGS-63	0	0	0	1	1	1	0	0	0	0	0	0	0
PGS-64	0	0	0	1	1	1	0	0	0	0	0	0	0
PGS-65	0	0	0	1	1	1	0	0	0	0	0	0	0
PGS-66	0	0	0	1	1	1	0	0	0	0	0	0	0
PGS-67	0	0	0	1	1	1	0	0	0	0	0	0	0
PGS-68	0	0	0	1	1	1	0	0	0	0	0	0	0
PGS-69	1	1	1	1	1	1	1	1	1	0	0	0	0
PGS-70	0	0	0	1	1	1	0	0	0	0	0	0	0
PGS-71	0	0	0	1	1	1	0	0	0	0	0	0	0
PGS-72	0	0	0	1	1	1	0	0	0	0	0	0	0
PGS-73	0	0	0	1	1	1	0	0	0	0	0	0	0
PGS-74	0	0	0	1	1	1	0	0	0	0	0	0	0
PGS-75	0	0	0	1	1	1	0	0	0	0	0	0	0
PGS-76	0	0	0	1	1	1	0	0	0	0	0	0	0
PGS-77	0	0	0	1	1	1	0	0	0	0	0	0	0
PGS-78	0	0	0	1	1	1	0	0	0	0	0	0	0
PGS-79	0	0	0	1	1	1	0	0	0	0	0	0	0
PGS-80	0	0	0	1	1	1	0	0	0	0	0	0	0
PGS-81	0	0	0	1	1	1	0	0	0	0	0	0	0
PGS-82	0	0	0	1	1	1	0	0	0	0	0	0	0
PGS-83	0	0	0	1	1	1	0	0	0	0	0	0	0
PGS-84	0	0	1	1	1	1	0	0	0	0	0	0	0
PGS-85	1	1	1	1	1	1	1	1	1	0	0	0	0
PGS-86	0	0	0	1	1	1	0	0	0	0	0	0	0
PGS-87	0	0	0	1	1	1	0	0	0	0	0	0	0
PGS-88	0	0	0	1	1	1	0	0	0	0	0	0	0
PGS-89	0	0	0	1	1	1	0	0	0	0	0	0	0
PGS-90	0	0	0	1	1	1	0	0	0	0	0	0	0
PGS-91	0	0	0	1	1	1	0	0	0	0	0	0	0
PGS-92	0	0	0	1	1	1	0	0	0	0	0	0	0
PGS-93	0	0	0	1	1	1	0	0	0	0	0	0	0
PGS-94	0	0	0	1	1	1	0	0	0	0	0	0	0
PGS-95	0	0	0	1	1	1	0	0	0	0	0	0	0
PGS-96	0	0	0	1	1	1	0	0	0	0	0	0	0
PGS-97	0	0	0	1	1	1	0	0	0	0	1	0	0
PGS-98	0	0	1	1	1	1	0	0	0	0	0	0	0
PGS-99	1	1	1	1	1	1	1	1	1	0	0	0	0
PGS-100	0	0	0	1	1	1	0	0	0	0	0	0	0
PGS-101	0	0	0	1	1	1	0	0	0	0	0	0	0
PGS-102	1	1	1	1	1	1	1	1	1	0	0	0	0
PGS-103	0	0	0	1	1	1	0	0	0	0	0	0	0
PGS-104	0	0	0	1	1	1	0	0	0	0	0	0	0
PGS-105	0	0	0	1	1	1	0	0	0	0	0	0	0
PGS-106	0	0	0	1	1	1	0	0	0	0	0	0	0
PGS-107	0	0	0	1	1	1	0	0	0	0	0	0	0
PGS-108	0	0	0	1	1	1	0	0	0	0	0	0	0
PGS-109	0	0	0	1	1	1	0	0	0	0	0	0	0
PGS-110	0	0	0	1	1	1	0	0	0	0	0	0	0
PGS-111	0	0	0	1	1	1	0	0	0	0	0	0	0
PGS-112	0	0	0	1	1	1	0	0	0	0	0	0	0
PGS-113	0	0	0	1	1	1	0	0	0	0	0	0	0
PGS-114	0	0	0	1	1	1	0	0	0	0	0	0	0
PGS-115	0	0	0	1	1	1	0	0	0	0	0	0	0
PGS-116	0	0	0	1	1	1	0	0	0	0	0	0	0
PGS-117	1	1	1	1	1	1	1	1	1	0	0	0	0
PGS-118	1	1	1	1	1	1	1	1	1	0	0	0	0
PGS-119	0	0	0	1	1	1	0	0	0	0	0	0	0
PGS-120	0	0	0	1	1	1	0	0	0	0	0	0	0
PGS-121	0	0	0	1	1	1	0	0	0	0	0	0	0
PGS-122	0	0	0	1	1	1	0	0	0	0	0	0	0
PGS-123	0	0	0	1	1	0	0	0	0	0	0	0	0
PGS-124	0	0	0	1	1	1	0	0	0	0	0	0	0
PGS-125	0	0	0	1	1	1	0	0	0	0	0	0	0
PGS-126	0	0	0	1	1	1	0	0	0	0	0	0	0
PGS-127	1	1	1	1	1	1	1	1	1	1	0	0	0
PGS-128	0	0	0	1	1	1	0	0	0	0	0	0	0
PGS-129	0	0	0	1	1	1	0	0	0	0	0	0	0
PGS-130	0	0	0	1	1	1	0	0	0	0	0	0	0
PGS-131	0	0	0	1	1	1	1	1	1	1	0	0	0
PGS-132	0	0	0	1	1	1	0	0	0	0	0	0	0
PGS-133	1	1	1	1	1	1	1	1	1	1	0	0	0

*Antibiotic abbreviations are as follows: **AMP**, Ampicillin; **AMX**, Amoxicillin; **CHL**, Chloramphenicol; **CIP**, Ciprofloxacin; **PEF**, Peflox; **NAL**, Nalidixic Acid; **SMX**, Sulfamethoxazole; **TMP**, Trimethoprim; **SXT**, Cotrimoxazole; **TCY**, Tetracycline; **AZM**, Azithromycin; **CFM**, Cefixime; **CTR**, Ceftriaxone; **Zero**, Susceptible; **One**, Resistance.

### Chloramphenicol resistance

Chloramphenicol resistance determinants were observed in 15.79% (21/133) strains by WGS (Table 1; Table S5 in Supplementary File 1). Nineteen non-susceptible strains harboured *catA1* gene which encodes chloramphenicol acetyltransferase enzyme causing chloramphenicol resistance by chemical modification of the drug molecule, whereas sixteen isolates harboured the *catI* genes. Our findings are consistent with other studies reporting chloramphenicol susceptibility in *S. enterica*^4,32^. Antimicrobial resistance to chloramphenicol was 8.27% (11/133) by disk diffusion method. Similar findings have been reported by other studies where resistance gene carriage rate was higher than phenotypically reported resistance^42,43^. The sensitivity was 100% and specificity was 91.80% for phenicol resistance (Fig. 4; Table 3; Table S7 in Supplementary File 1).

### Co-trimoxazole resistance

Trimethoprim-sulfamethoxazole were considered in combination for treatment as the first-line drug, co-trimoxazole. Out of 133 strains, trimethoprim resistance determining genes were found in 15% isolates (20/133). The most prevalent *dfrA7* was observed in 18 isolates followed by *dfrA15* and *dfrA1* in three isolates. Likewise, gene *sul1* and *sul2*, encoding dihydropteroate synthases known to disseminate sulfamethoxazole resistance, were detected in overall 15.79% isolates (21/133), where 16 isolates harboured both *sul1* and *sul2* genes and separately *sul1* and *sul2* genes alone were observed in 3 and 2 strains, respectively (Table 1; Table S5 in Supplementary File 1). Overall, antimicrobial resistance to co-trimoxazole was detected in 21 (15.79%) strains by WGS as compared to 12 (9.02%) by phenotypic method. The trimethoprim and sulfamethoxazole resistance markers individually were perceived in two isolates. These results are in concordance with previous findings from India which reported antimicrobial resistance in 6% and 5% of typhoidal salmonella isolates^4,32^. The sensitivity was 100% and specificity was 99.56% for co-trimoxazole (Fig. 4; Table 3; Table S7 in Supplementary File 1).

### Fluoroquinolones resistance

Molecular determinants of resistance to fluoroquinolone including ciprofloxacin and pefloxacin antibiotics encoded by *gyr*A*, gyr*B*, par*C, and *par*E genes were detected in 96.99% strains (129/133) by WGS. Mutations in *gyrA, parC, parE* and *gyr*B was observed in 94.74%, 21.05%, 5.26% and 2.26% of strains, respectively (Tables 1–2; Table S5-S6 in Supplementary File 1). The identified genes were associated with mutations in Quinolone Resistance Determining Region of DNA gyrase enzyme, the binding site for fluoroquinolone. Antimicrobial resistance to fluoroquinolones was 97.74% (130/133) by both disc diffusion and E-test method. MIC distribution ranged between 2–24 mg/L and peaked at 12 mg/L. A gradual increase in decreased susceptibility to ciprofloxacin was demonstrated in a longitudinal analysis of past 20 years^44^. DNA Gyrase A mutations at position 83 (Ser-83→Phe and Ser-83→Tyr) are the most prevalent resistance mechanisms for fluoroquinolone in India^45^, followed by Ser-80→Ile substitution in *parC* gene. We also found one fluoroquinolone susceptible strains with mutation in *gyrA* gene which might be occurring due to bacterial promoter modifications leading to the overexpression of other DNA topoisomerases. Highly non-susceptible strains (with ciprofloxacin MIC > 8 mg/L) were found to be double or triple mutants with mutations in *gyrA*83, *gyrA*87 and *parC*80. Strains with moderate resistance to ciprofloxacin possessed single mutations in DNA *gyrA* gene at Ser83 position. The observed sensitivity was 99.23% and specificity was 100% for both ciprofloxacin and peflox resistance (Fig. 4; Table 3; Table S7 in Supplementary File 1).

**Table 2:** Chromosomal point mutations associated with the quinolone resistance-determining region (QRDR) and azithromycin resistance for *S*. Typhi.

AMR gene	AMR SNP’s	Resistance type	Frequency
gyrA	D87N, S83Y, D87Y, S83F, D87G	fluoroquinolone	94.74%
gyrB	S464F, A574V	fluoroquinolone	6.77%
parC	E84G, E84K, S80I	fluoroquinolone	21.05%
parE	A364V, D420N, L416F	fluoroquinolone	11.28%
acrB	R717Q	azithromycin	0.75%

**Table 3:** Genotypes-phenotypes correlation prediction for all 133 *Salmonella* isolates in the study.

Antibiotic	Phenotypically Non-susceptible		Phenotypically Susceptible		Sensitivity (%)	Specificity (%)
	Genotypically Non-susceptible	Genotypically Susceptible	Genotypically Non-susceptible	Genotypically susceptible		
**Ciprofloxacin**	129	1	0	3	99.23	100
**Pefloxacin**	129	1	0	3	99.23	100
**Amoxicillin**	16	0	5	112	100	95.73
**Chloramphenicol**	11	0	10	112	100	91.80
**Co-trimoxazole**	12	0	9	112	100	92.56
**Cefixime**	0	0	0	133	100	100
**Ceftriaxone**	0	0	0	133	100	100
**Azithromycin**	1	1	0	131	50	100
**Overall**					**93.56**	**97.51**

### Cephalosporins resistance

- Antimicrobial susceptibility to antibiotics, cefixime and ceftriaxone, observed for all strains is consistent with other studies from India^1,32^. Resistance determinants were also not found for cephalosporins (Table 1; Table S5 in Supplementary File 1). Though all the strains were susceptible, however, a gradual increase in median MIC values was perceived over a time period. This clearly raises an alarm towards the judicial use of these antibiotics.

### Azithromycin resistance

Only 0.75% non-susceptible strains (1/133) to azithromycin were observed by WGS as compared to 1.50% (2/133) strains by phenotypic method. The non-susceptible strains harboured mutations in the *acrB* efflux pump regulator genes that have been known to confer macrolide resistance^46^. In this study, we detected R717Q mutation on *AcrB* efflux pump as a cause of azithromycin resistance in *S*. Typhi^11^ (Tables 1–2; Table S5-S6 in Supplementary File 1). AcrB-R717Q precisely leads to increased macrolide resistance. This study indicated that most of the *S*. Typhi strains were susceptible to azithromycin. These strains were screened separately for presence of macrolide resistance determinants (*mphA, ermA, ereA, ereB, mefA* and *msrA* genes) by PCR and presence of mutations in *rlpD* and *rlpV*, where also no macrolide resistance mechanism was observed^46^. The sensitivity was 50% and specificity was 100% for azithromycin resistance (Fig. 4; Table 3; Table S7 in Supplementary File 1).

### Genotypic resistance to other antimicrobial agents

*S*. Typhi can demonstrate resistance to multiple antibiotics by acquiring new resistance genes through horizontal genes transfer (HGT). The acquired antimicrobial resistance genes including *aac(6’)-Iaa, AAC(6’)-Iy, aadA1, aph(3”)-Ib, aph(6)-Id, strA*, and *strB* that provided resistance to aminoglycosides were observed in 100% (133/133) isolates (Table 1; Table S5 in Supplementary File 1). Tetracycline resistance encoded by *tet(A), tet(B),* and* tet(R)* genes for tetracycline efflux pumps were detected mainly in 9.02% strains (12/133) of *S*. Typhi (Table 1; Table S5 in Supplementary File 1). In addition, *S*. Typhi isolates harboured the genes *baeR, emrb, H-NS, marA, mdfA, mdtK, msbA, acrA, emrR, kpnE, kpnF, marR, sdiA, crp, soxR*, and *soxS* that could confer multidrug resistance and were detected in all 133 strains (Table S5 in Supplementary File 1). The mdsABC complex, a multidrug transporter of *Salmonella*, comprising *mdsA, mdsB*, and *mdsC* units was observed in every single isolate. The mdsABC complex is recognized to contribute resistance against a diverse set of drugs and toxins^47^. The identified multi-efflux pump* mdtK* gene, conferring resistance against the drugs, acriflavin, doxorubicin and norfloxacin, was observed in 100% (133/133) of the isolates^48^. The gene, *sdiA*, a multi-drug resistance pump regulator for AcraB, was also present in 100% (133/133) of the isolates^49^. The pathogenicity and resistance profile of the various Salmonella isolates can be attributed to the presence of identified genes.

### MLST and cgMLST-based phylogenetic analysis

Whole genome sequencing-based *in silico* multi-locus sequence typing (MLST) profile disclosed low genetic variation in housekeeping genes (*aroC, dnaN, hemD, hisD, purE, sucA*, and *thrA*) among 133 *Salmonella* isolates. Two different sequence types (STs) including ST1 and ST2, irrespective the year of isolation were observed^49^. ST1 was the predominant type, accounting for 81.95% (109/133) of examined strains, whereas ST2 was observed in 17.29% (23/133) of the strains (Table 4; Table S8 in Supplementary File 1). In addition, one novel ST, *aroC1* was identified which consists of a missense substitution (C– > T) compared to the predominant *aroC1* allele present in almost all *S*. Typhi strains. MLST analysis revealed the presence of ST1 in all strains, irrespective of the geographical location, whereas ST2 was completely absent in strains isolated at Chennai and Wardha city of India. We next investigated the epidemiological outbreak using the phylogenetic tree based on gene-by-gene-based core genome MLST (cgMLST) method. The analysis yielded a total of 3002 core loci, where the number of successfully called alleles per isolate ranged from 2664 (88.74%) to 2706 (90.14%) depending on the gene. For high-resolution subtyping, core-genome sequence types (cgST) were considered, where the allele matches in cgST per isolate ranged from 2634 (87.74%) to 2699 (89.91%). The cgST-based tree showed that isolates with identical ST1 (119 strains) and novel ST (1 strain) were clustered in one group, whereas isolates with ST2 (23 strains) were separated into outlier cluster (Fig. S2 in Supplementary File 3; Table S9 in Supplementary File 1). Inter-strain diversity among all isolates yielded the Simpson’s diversity index (SDI) equal to 0.51. The result demonstrates a lower level of genetic diversity and epidemiological links among isolates of *S*. Typhi.

**Table 4:** Genotype-associated sequence types (ST), mutation patterns in the genes encoding DNA gyrase (*gyrA* and *gyrB*) or topoisomerase (*parC* and *parE*) and known plasmid Inc types in *S*. Typhi.

Genotypes	No. of isolates (%)	MLST ST	gyrA D87N	gyrA S83Y	gyrA D87Y	gyrA S83F	gyrA D87G	gyrB S464F	parC S80I	parC E84G	parC E84K	parE D420N	parE L416F	Inc Types
2.2	4 (3.01)	2	0	0	0	1	0	0	0	0	0	0	0	
2.5	3 (2.26)	2	0	0	0	1	1	0	0	0	0	0	0	
3	1 (0.75)	1	0	0	0	1	0	0	0	0	0	0	0	
3.3	4 (3.01)	2	0	0	0	1	0	0	0	0	0	0	0	IncFIB(pHCM2)
2.2.2	2 (1.50)	2	0	1	0	0	0	0	0	0	0	0	1	
2.2.4	1 (0.75)	2	0	1	0	0	0	0	0	0	0	0	0	
3.3.1	6 (4.51)	2	0	1	0	0	0	0	0	0	0	0	1	
3.3.2	3 (2.26)	2	0	1	1	0	0	0	0	0	0	0	0	
4.3.1	19 (14.29)	1	0	1	0	1	1	1	0	0	0	1	0	IncFIA(HI1); IncHI1A/IncHI1B(R27)
4.3.1.1	18 (13.53)	1	0	1	0	1	1	1	0	0	1	0	0	IncHI1A/IncHI1B(R27); IncFIB(pHCM2); Col(BS512)
4.3.1.2	72 (54.14)	1	1	1	0	1	0	0	1	1	1	0	0	IncFIB(pHCM2); IncN; p0111; IncHI1A/IncHI1B(R27)

### Population structure and QRDR mutation patterns

The genotyphi classification according to the scheme of Wong *et al*.^50^, revealed 11 distinct genotypes (2.2, 2.2.2, 2.2.4, 2.5, 3, 3.3, 3.3.1, 3.3.2, 4.3.1, 4.3.1.1, and 4.3.1.2) signifying the diverse population structure in India (Table 4; Table S8 in Supplementary File 1).The majority (81.95%) of the isolates belonged to H58 clade including H58 lineage (genotype 4.3.1), H58 lineage I (genotype 4.3.1.1) and H58 lineage II (genotype 4.3.1.2)^23,51–54^. The H58 lineage (genotype 4.3.1) was present in 14.29% (19/133) of *Salmonella* isolates obtained in India from January 1993 to December 2016 and probably emerged from South Asia and East Africa in the initial 1990s^54,55^. H58 lineage I (genotype 4.3.1.1) was present in 13.53% (18/133) of the isolates, whereas H58 lineage II (genotype 4.3.1.2) was dominant in the majority of the isolates (70/133, 52.63%). The H58 lineage II isolates appeared to be dominant in Nepal, India and Pakistan^12,51,52^, whereas it was rare in nearby country Bangladesh^56^. In contrast, H58 lineage I (genotype 4.3.1.1) isolates were dominant (31.2%) in Bangladesh compared to India (13.53%). The genotypes 3, 3.3, 3.3.1 and 3.3.2 were collectively observed for 10.53% of *Salmonella* isolates, whereas genotypes 2.2, 2.2.2 and 2.2.4 were found in 7.52% of isolates. The reference-based (Fig. S3 in Supplementary File 4) and recombination free (Fig. S4 in Supplementary File 5) phylogenetic analysis was carried out separately to locate outbreak clusters based on SNPs shared among *S*. Typhi isolates collected from diverse geographical locations^55^. The tree showed that the strains were mainly grouped into two clusters, where 17.29% of strains (23/133) clustered close together with a distinct segregation from others strains (109/133). The phylogenies revealed that 93.39% of the reference genome (*S*. Typhi CT18) was covered in all isolates. The minimum and maximum SNPs differences in comparison to reference genome was observed for PGS-123 (275 SNPs) and PGS-66 (521 SNPs), respectively (Table S10 in Supplementary File 1). SNP tree was further explored to investigate the distribution and mutation patterns in the genes encoding DNA gyrase (*gyrA* and *gyrB*) or topoisomerase (*parC* and *parE*) that reside in the quinolone resistance-determining region (QRDR). The overall high genotypic (96.99%) and phenotypic (97.74%) resistance to ciprofloxacin and pefloxacin was observed owing to QRDR mutants in *Salmonella* isolates (Fig. 4; Table S6 in Supplementary File 1). This is similar to the previously reported cases where the QRDR mutants were dominant in India (97%), Bangladesh (94%), Nepal (66%), Pakistan (94%), Myanmar (100%), Uganda (100%), and Nigeria (50%)^54^. The close examination of the tree exposed the genotype-based clustering in *S*. Typhi. The minimum and maximum numbers of SNP differences within the cluster strains were observed to be low as compared to strains belonging to different clusters and reference strain. The isolates belonging to genotype 3.3.2 were clustered in two clades, each with a different mutation (S83Y; D87Y) in the Quinolone Resistance Determining Region (QRDR) of gene *gyrA* as conveyed in Bangladesh^56^. QRDR double mutation in *gyrA* (S83F) and *parE* (L416F) was commonly observed in the cluster of genotype 3.3.1 which is linked with travel to West Africa^54^. QRDR triple mutants, resistant to ciprofloxacin was observed in genotype 4.3.1 (*gyrA*_S83Y, S83F, D87G; *gyrB*_S464F; *parE*_D420N) and genotype 4.3.1.1 (*gyrA*_S83Y, S83F, D87G; *gyrB*_S464F; *parC*_E84K) which were known to be associated with travel to India^54^. Triple mutant (*gyrA*-S83F, *gyrA*-D87N and *parC*-S80I) conferred resistance to ciprofloxacin in *S*. Typhi were common in the cases reported with travel to India, Pakistan and Nepal. The mutation in *gyrA* (S83F) was commonly observed in seven genotypes 2.2, 2.5, 3, 3.3, 4.3.1, 4.3.1.1, and 4.3.1.2. Similarly, shared mutation in *gyrA* (S83Y) was observed between seven genotypes namely 2.2.2, 2.2.4, 3.3.1, 3.3.2, 4.3.1, 4.3.1.1, and 4.3.1.2. The mutation frequency of *gyrA*_S83F (n = 74; 55.64%) observed in our data was accordance with previously reported cases of travel to South Asia including India (n = 71), Pakistan (n = 134), and Bangladesh (n = 25), and in contrast with travel history to Central Asia (n = 1), Europe (n = 1), Middle East (n = 1), and South America (n = 1)^54^. The mutation frequency of *gyrA*_S83Y (n = 48; 36.09%) was similar with previously discussed cases of travel to India (n = 56). The shared QRDR mutations between *S*. Typhi lineages in the phylogeny indicated that they might have inherited from a shared common ancestor. Genotype-associated mutation patterns are given in Table 4 and Fig. S4 in Supplementary File 5.

## Methods

### Study design

This study was conducted on clinical strains isolated from the blood culture of patients presented with typhoid fever from January 1993 to December 2016. We used blood culture isolates from bacteriology laboratory, A.I.I.M.S., New Delhi, where the samples were sent as a part of routine diagnosis. All the procedures were performed as per CLSI guidelines. All the experiments and data analysis were completed at All India Institute of Medical Science (A.I.I.M.S.), New Delhi.

### Bacterial isolates

Clinical isolates of *S*. Typhi were revived from the archived cryopreserved collection of blood culture isolates and identified by standard biochemical and serological methods as described earlier^57^. The collection comprised 686 strains from which 133 strains of *S*. Typhi were selected using random number generator function in Stata v.14.1 (StataCorp, College Station, TX, USA)^58^. The strains were serially named as PGS1 (Pan Genome Study) to PGS133 for simplicity in data analysis.

### Whole genome sequencing

Genomic DNA isolated from freshly grown overnight culture (*QIAamp* DNA minikit; *Qiagen*, Germany) was quantified using Qubit fluorometer (Life Technologies, USA). Sequencing library preparation was by Illumina Nextera XT DNA sample preparation kit (Illumina, USA). Genomic DNA samples were fragmented using Covaris M-series (M220) at temperature of 5.5 to 6 °C for 40 seconds. DNA fragments were end repaired using dA bases before ligation with Illumina indexed adapters, amplified for 10 cycles of PCR and sequenced employing v2 and v3 chemistry with paired-end 2 × 151 bp reads on Illumina MiSeq (Illumina, USA). Output data files were de-multiplexed and transformed with Casava v.1.8.2. into FASTQ files (Illumina, Inc, USA).

### Preprocessing, genome assembly and analysis

The selected sequence paired-end reads from FastQC v0.11.4^59^ were pre-processed and assembled *de novo* with A5-miseq pipeline^60^. Sequence adapters and low-quality (<Q30) regions were filtered with trimmomatic v0.36^61^. Read errors were corrected by SGA’s k-mer-based error correction algorithm^62^. Paired and unpaired reads were assembled utilizing IDBA-UD algorithm and quality of genome assembly evaluated by QUAST (http://quast.sourceforge.net/quast)^63^. The redundant homologues with identity cut-off of 0.9 were removed from assembled *de novo* contigs by CD-HIT^64^. The assembled genomes were aligned to a reference genome of *S*. Typhi to avoid the risk of cross-contamination. The assembled bacterial genomes were annotated with Prokka v1.12^65^ (http://www.vicbioinformatics.com/software.prokka.shtml). The total numbers of coding sequence regions (CDS) in annotated genomes were compared among all strains to filter out outlier strains.

### Pan-genome construction

To identify strain-specific genomic features and genomic diversity among *S*. Typhi isolates, the pan-genome was constructed using computational pipeline BPGA^66^. The entire set of proteins served as input for the BPGA analysis. The clustering of genes into families was generated with USEARCH^67^ with 90% sequence identity as a cutoff. To reduce the impact of outliers, the core-and pan-genome size was determined for two isolates at first, and then the remaining isolates were added iteratively till the exact contaminant strain liable for shrinking the core-genome size was detected. The outliers were excluded in the final version of pan-genome. In order to evade any bias during the sequential inclusion of genomes, random sequence/order of genomes permutations were performed. The pan-genome functional analysis module of BPGA was used to assign Cluster of Orthologous Genes (COG)^35^ and Kyoto Encyclopedia of Genes and Genomes (KEGG)^36^ classes to the core, accessory, and unique gene families. The results were plotted with Gnuplot and PanGP^68,69^.

### Identification of resistance determinants

The resistance genes in the assembled *Salmonella* genomes were predicted through the resistance gene identifier (RGI) from the Comprehensive Antibiotic Resistance Database (CARD, available at https://card.mcmaster.ca/analyze/rgi)^70^ and Pathogenwatch from the Center for Genomic Pathogen Surveillance (CGPS, available at https://pathogen.watch) databases of antimicrobial resistance genes^71^. We used cut-off criteria of ≥ 50% sequence identity and ≥70% query coverage. RGI (RGI 4.2.0, CARD 2.0.3) prediction of resistome was determined based on homology and SNP models, where the “perfect and strict hits only” criteria were chosen for the prediction. ResFinder webserver 3.0 (https://cge.cbs.dtu.dk/services/ResFinder/) was used to pinpoint the acquired antimicrobial resistance genes and genes associated with chromosomal point mutations^72^.

### Phenotypic antimicrobial susceptibility determination

The testing for antimicrobial susceptibility was through Kirby–Bauer disc diffusion assay for chloramphenicol (CHL; 30 µg), ampicillin (AMP 10 µg), trimethoprim-sulfamethoxazole (TMP/SMX; 1.25/23.75 µg) ciprofloxacin (CIP; 5 µg), pefloxacin (PFX; 5 µg), cefixime (CFM; 5 µg), ceftriaxone (CTR; 30 µg), and azithromycin (AZM; 15 µg) as per CLSI guidelines^73^. Ampicillin disk diffusion test was used to predict the antimicrobial susceptibility results for amoxicillin as recommended by current CLSI guidelines^73^. Minimum inhibitory concentration (MIC) was determined for ciprofloxacin, ceftriaxone and azithromycin by E-test method (E. test, BioMerieux, France). Quality control for antimicrobial susceptibility was done using *Escherichia coli* ATCC 25922.

### Correlation of susceptibility genotypes and phenotypes

Overall 1064 phenotypic data analysis points detected from 133 strains through phenotypic antimicrobial susceptibility testing were correlated with the existence of corresponding plasmid mediated resistance gene(s) as well as structural gene mutations. Phenotypically intermediate and resistant strains were together referred as non-susceptible in this investigation. Considering phenotypic results as the reference outcome, sensitivity of genomic method of antimicrobial susceptibility determination was calculated by dividing the number of genotypically non-susceptible isolates by the overall sum of isolates displaying phenotypic resistance. Specificity was determined by dividing the number of genotypically susceptible isolates by total number of phenotypically susceptible isolates.

### Phylogenetic tree based on cgMLST and SNPs

Whole-genome sequencing (WGS)-based *in silico* multi-locus sequence typing (MLST) of *Salmonella* strains were performed using MLST 2.0 server^74^. The gene-by-gene-based core genome MLST (cgMLST) was completed using cgMLSTFinder (version 1.1) server^75^, whereas referenced-based SNP tree using maximum likelihood method was constructed by CSI phylogeny (version 1.4) server^76^ from the Centre for Genomic Epidemiology (www.genomicepidemiology.org). The CSI phylogeny build phylogenetic tree is based on the concatenated alignment of the high-quality SNPs, where SNPs were filtered out if the depth at the SNP position was not at least 10x or at least 10% of the average depth for the particular genome mapping. SNPs were also filtered out if the mapping quality was below 25 or the SNP quality was below 30. The genotypic profile and recombination free SNP tree was built using Pathogenwatch from the Center for Genomic Pathogen Surveillance (CGPS, available at https://pathogen.watch). The phylogenetic tree was visualized using FigTree v1.4.3 (http://tree.bio.ed.ac.uk/software/figtree/)^77^ and iTOL (https://itol.embl.de/)^78^.

### Ethics declarations

The protocol for this study was approved (IEC/NP-283-2012) by the Ethics Committee, All India Institute of Medical Science, New Delhi, India.

## Conclusions

*Salmonella* Typhi continues to pose a major challenge in treatment as it causes invasive infections and acquires antimicrobial resistance (AMR) genes to become non-susceptible to available drugs^79^. As the pipeline for new antityphoidal drugs are exhausted, it has become imperative to explore whole genomes to understand the characteristics and search for novel diagnostic targets. Findings from this study revealed a high correlation between the phenotypes and their corresponding genotypes. The pan-genome analysis revealed the characteristics of gene pool shared by clinical *Salmonella* Typhi isolates, indicating that the core genes were enriched in metabolism related function whereas accessory genes were majorly implicated in pathogenesis and antimicrobial resistance mechanisms. The cgMLST and SNP-based phylogenetic analysis revealed lower level of genetic diversity where a larger of the strains belonged to the same outbreak and few were separated into outlier cluster. This work overall highlights the importance of WGS-based resistance gene screening for the tracking of AMR mechanisms in *S*. Typhi and thus can^66^ contribute to the battle against the expanding AMR threat worldwide.

## Supplementary information

Supplementary Tables.

Supplementary information 2.

Supplementary information 3.

Supplementary information 4.

Supplementary information 5.

## Data Availability

WGS data of all 133 Salmonella isolates have been submitted to the National Center for Biotechnology Information (NCBI; https://submit.ncbi.nlm.nih.gov/subs/bioproject/) under BioProject accession number PRJNA564922.
