# Supplementary figures and images for: Genomic profiling of antimicrobial resistance genes in clinical isolates of Salmonella Typhi from patients infected with Typhoid fever in India
Source: Sci Rep. 2020 May 19;10:8299. doi: 10.1038/s41598-020-64934-0 (PMC7237477; doi:10.1038/s41598-020-64934-0)

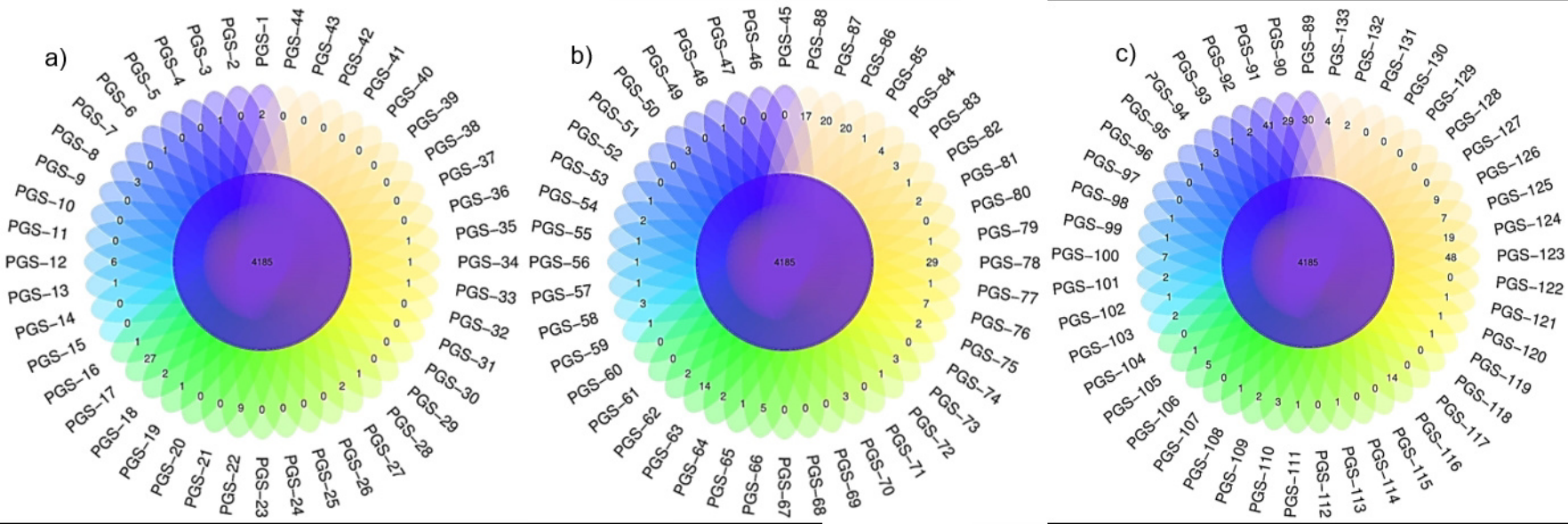

Supplement: Supplementary file 2 — Supplementary information 2. [file 41598_2020_64934_MOESM2_ESM.pdf]

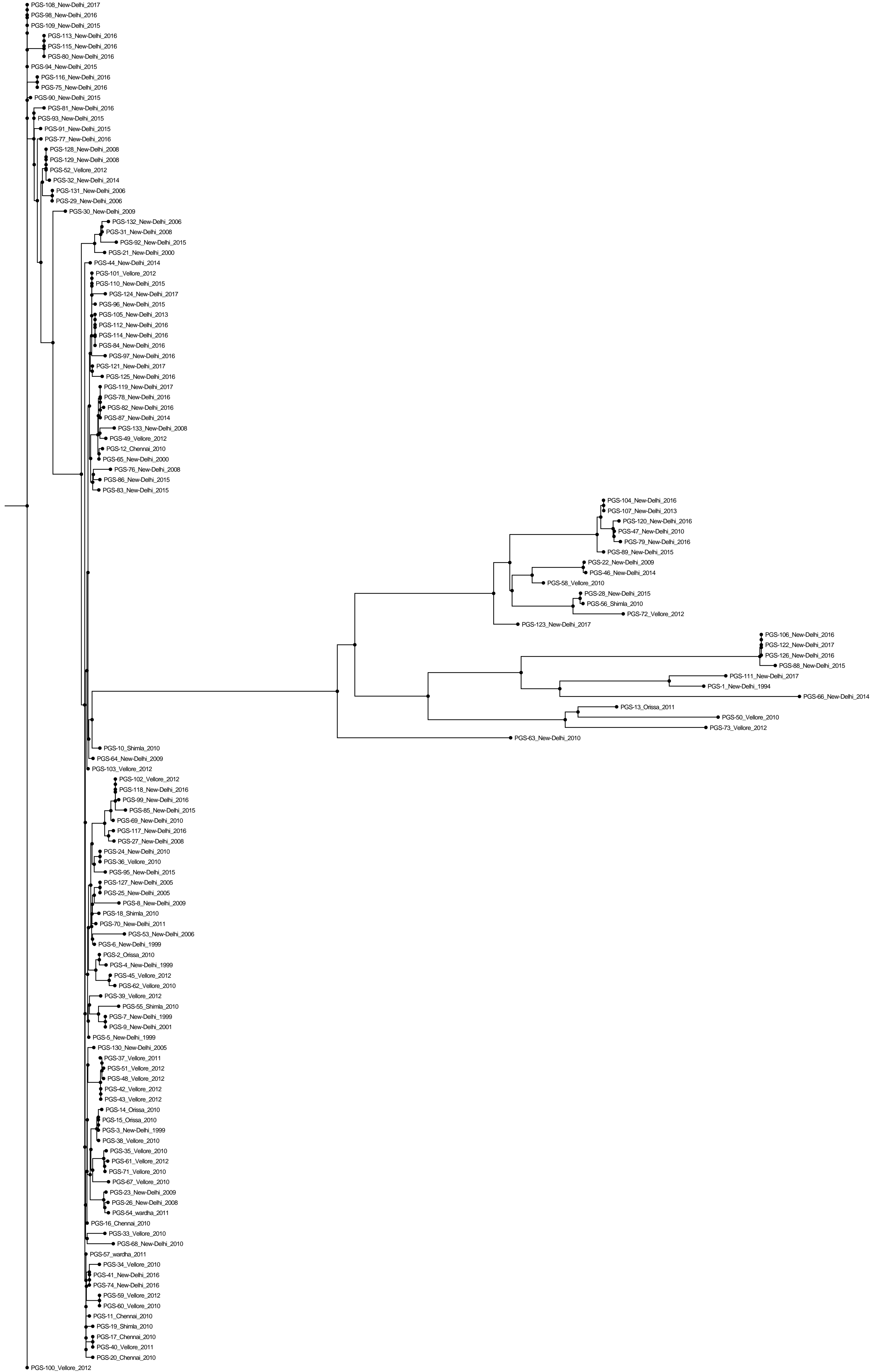

Supplement: Supplementary file 3 — Supplementary information 3. [file 41598_2020_64934_MOESM3_ESM.pdf]

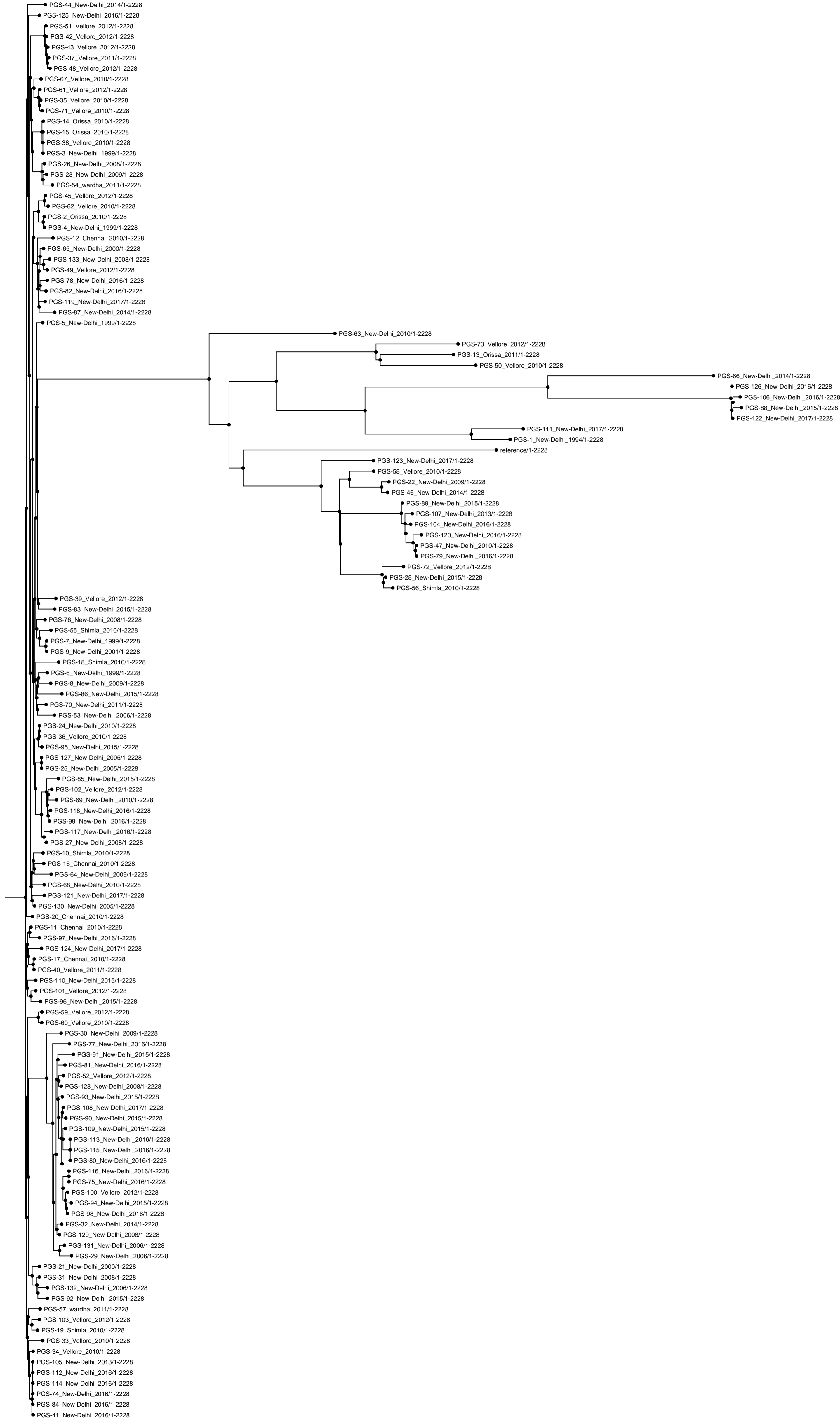

0.05

Supplement: Supplementary file 4 — Supplementary information 4. [file 41598_2020_64934_MOESM4_ESM.pdf]

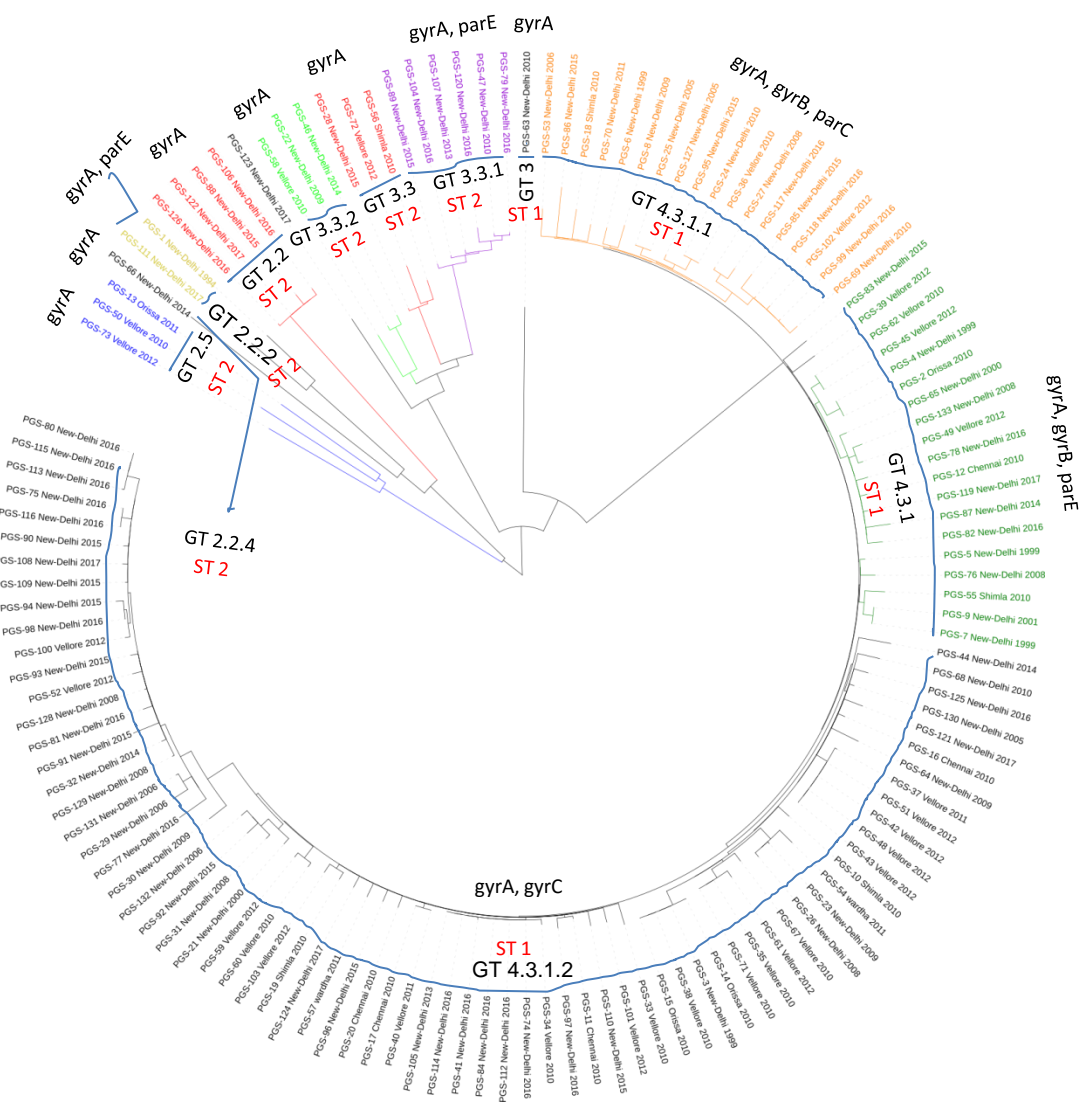

Supplement: Supplementary file 5 — Supplementary information 5. [file 41598_2020_64934_MOESM5_ESM.pdf]
